# Adaptive propensity score procedure improves matching in prospective observational trials

**DOI:** 10.1186/s12874-019-0763-3

**Published:** 2019-07-16

**Authors:** Dorothea Weber, Lorenz Uhlmann, Silvia Schönenberger, Meinhard Kieser

**Affiliations:** 1 Institute of Medical Biometry and Informatics, University of Heidelberg, Marsilius Arkaden, Im Neuenheimer Feld 130.3, Heidelberg, 69120 Germany; 2 Department of Neurology, Heidelberg University Hospital, Im Neuenheimer Feld 400, Heidelberg, 69120 Germany

**Keywords:** Adaptive design, Clinical Trials, Sample size recalculation, Matched cohort, Prospective matching

## Abstract

**Background:**

Randomized controlled trials are the gold-standard for clinical trials. However, randomization is not always feasible. In this article we propose a prospective and adaptive matched case-control trial design assuming that a control group already exists.

**Methods:**

We propose and discuss an interim analysis step to estimate the matching rate using a resampling step followed by a sample size recalculation. The sample size recalculation is based on the observed mean resampling matching rate. We applied our approach in a simulation study and to a real data set to evaluate the characteristics of the proposed design and to compare the results to a naive approach.

**Results:**

The proposed design achieves at least 10% higher matching rate than the naive approach at final analysis, thus providing a better estimation of the true matching rate. A good choice for the interim analysis seems to be a fraction of around $\frac {1}{2}$ to $\frac {2}{3}$ of the control patients.

**Conclusion:**

The proposed resampling step in a prospective matched case-control trial design leads to an improved estimate of the final matching rate and, thus, to a gain in power of the approach due to sensible sample size recalculation.

**Electronic supplementary material:**

The online version of this article (10.1186/s12874-019-0763-3) contains supplementary material, which is available to authorized users.

## Background

Randomized controlled designs are the gold-standard for clinical trials. The advantage of a random allocation of patients to treatment and control group is the comparability of patient groups. However, there are situations where a randomized trial is not applicable, for instance, due to ethical concerns or practical reasons, but an observational trial is possible [1, 2]. An observational single-arm study might be an option, but has the disadvantage that a direct comparison to placebo or the standard therapy is not possible. When data on the control recruited within an earlier study is available, an alternative way would be to use this external control group for comparison. Naively comparing study arms of different trials may lead to severe bias due to differences in patient characteristics. The lack of comparability can be addressed by matching procedures as, for example, Optimal Matching or Propensity Score Matching [3, 4]. These methods aim to balance the groups by the variables considered within the matching procedure. Combining a prospective single-arm study with an external control group under the usage of a matching approach is called a prospective matched case-control trial [5].

One essential part in planning a clinical trial is the calculation of the required sample size. In a prospective matched case-control trial, sample size calculation is not straightforward. The aim is to find an appropriate matching partner for as many patients of the already recruited study arm as possible. Usually it cannot be expected to find a matching partner for all patients in the control group when recruiting just the same number of patients in the treatment arm. Therefore, published trials fixed an additional percentage of intervention patients; for example, the trial of Charpentier et al. [5] recruited 1.3 times the number of control patients. In case a lower or higher number of patients can be matched to one of the controls than expected, the sample size would be too small or more patients than needed are recruited. In the following, the fraction of patients matched to one of the controls is called matching rate.

This work is motivated by a real data example, the KEEP SIMPLEST trial [6]. The trial aimed to compare aspects of periinterventional management in acute ischemic stroke (AIS) patients treated according to a new SOP (standard operating procedure) with patients having been randomized into the conscious sedation group of the SIESTA trial [7]. A randomized controlled trial was not applicable, because the early stage application of the new method cannot be reproduced. Therefore, a prospective matched case-control design was planned. The matching rate was unknown and as in most other trials most likely less than 100%, meaning that recruiting just the same number of patients as in the external control arm will not result in a situation where a matching partner is found for all patients in the control group. A possibility to address this uncertainty concerning the matching rate could be to perform an interim analysis to estimate the actual matching rate. The results of the interim analysis are then used to recalculate the sample size. This leads to an adaptive matched case-control design.

The recalculation might be done by using all available patients in the matching procedure (at interim and final analysis, respectively). Based on the matching rate observed in the interim analysis, the sample size is recalculated. This strategy will be called the *naive method*. In practice, we expect there may occur a potential overestimation of the matching rate. In consequence, a smaller number of patients than necessary is recruited after interim analysis and therefore, a smaller matching rate is achieved at the final analysis. To avoid overestimation, we propose a method for calculating the matching rate at interim analysis which is characterized by a resampling step and recalculation of the sample size based on the mean resampling matching rate. The time point of this interim analysis needs to be fixed at the beginning of the trial. We conducted a simulation study to investigate the operational characteristics of the proposed approach and to develop a recommendation for the time point of interim analysis.

In “Methods”, we explain the proposed method for calculating the sample size and the details of the conducted simulation study. Its results are described and followed by the application to the real data example in “Results”. The results are discussed and conclusions are drawn in “Discussion and conclusion”.

## Methods

We propose an adaptive design for recalculating the sample size in a prospective matched case-control trial which is characterized by a resampling approach and a propensity score matching step.

Within the suggested design, two matching steps are conducted. At interim analysis, the matching rate is determined and is used for recalculation of the sample size needed to find a matching partner to all control patients in the final analysis. The matching procedure at interim analysis is solely used for calculating the matching rate; the final 1:1 matches are determined in the final analysis. To find pairs of treated and control patients, the propensity score method by Rosenbaum and Rubin [8, 9] is used. Propensity score matching aims to minimize the influence of observed and considered baseline characteristics on the treatment effect [10]. The propensity score *e*(*X*) is the conditional probability of being assigned to the treated study arm given (relevant) confounders *X* [10]. Assuming that there are *n* patients included, the propensity score is defined as

$$e(X_{i}) = P(Z_{i} = 1 \mid X_{i}) \qquad i=1,\ldots, n,$$

where *Z*_*i*_∈{0,1} defines the group assignment and *X*_*i*_ is the vector of considered baseline characteristics.

The propensity score is estimated by using baseline characteristics as covariates in a logistic regression model with treatment status as outcome variable [9]

1 $$ logit(Z_{i}) = \beta_{0, gr} + \beta_{1, gr}X_{i1} + \beta_{2, gr}X_{i2} + \ldots\  $$

This model provides the propensity scores, the probability to be assigned to the treatment group. The treatment and control patients are matched according to the logit of the estimated propensity score

$$\ln\frac{e(X_{i})}{1-e(X_{i})}$$

by using some caliper width of these estimates [4]. Austin recommend a caliper width of 0.2 of the standard deviation of the logit of the propensity score [10].

The already recruited study arm includes *n*_*control*_ patients. To determine the matching rate at the interim analysis, we resample the control patients. In order to further avoid an overestimation of the matching rate at interim analysis equally sized groups are used for the matching procedure at interim analysis. That means, a sample of *n*_*treated,interim*_ is taken from the *n*_*control*_ patients without replacement. Using the sampled set of patients, the matching step is performed which is explained in the following. This resampling and the matching step at interim analysis are repeated *b* times.

We calculate the mean resampling matching rate $\overline {mr}$ and the lower limit of the 100·(1−*α*_*CI*_) % confidence interval (CI) (using *n*_*control*_)

2 $$ l_{mr} = \overline{mr} - \phi(1-\alpha_{CI}) \cdot \sqrt{\overline{mr}*(1-\overline{mr})/n_{control}}.  $$

The total number of patients needed in the treated group is estimated by

3 $$ n_{treated, final} = \frac{n_{control}}{l_{mr}}.  $$

In the following, this approach is called *resampling CI method*.

Another option would be to use a quantile of the distribution of the resampling matching rates which are independent of the number of patients in the control group. One would expect to observe a higher matching rate in trials with a large control arm, because a higher diversity of patients may be represented. Therefore, taking the number of control patients into account has the advantage of a smaller confidence interval for a larger number of control patients. For this reason, we stick with our proposed definition of the 100·(1−*α*_*CI*_)% CI.

Steps of the procedure at interim analysis:

Given entities:

- *b* the number of resampling steps.
- *n*_*control*_ the number of control patients in already recruited study arm.
- *n*_*treated,interim*_ the number of treated patients at interim analysis.
  Step 1 Repeat (a) - (d) *b* times:
    - Sample *n*_*treated,interim*_ patients without replacement out of the control group.
    - Calculate propensity scores for sampled control patients and treated patients.
    - Conduct a 1:1 matching according to the logit of the propensity scores.
    - Calculate the matching rate *mr*.
  Step 2 Calculate the mean matching rate $\overline {mr}$ of the *b* matching rates calculated in step 1.
  Step 3 Calculate the lower limit of the 100·(1−*α*_*CI*_)% confidence interval using formula (2).
  Step 4 Calculate the total number of treated patients needed for analysis as in formula (3).

We conducted a simulation study with 10,000 replications of each scenario, to assess the performance of our approach. First, we compare the resampling CI method for recalculating the sample size to the naive strategy. The second part covers the determination of the optimal time point for the interim analysis.

### Simulation setting

#### General setting

The chosen values for the involved parameters are simulated inspired by the clinical example (“Real data example” section). Some simplifications (e.g. less variables within the matching procedure) were made for the simulation study.

The outcome variable is assumed to be binary indicating some favourable event and the corresponding hypotheses are

$$\begin{array}{*{20}l} \mathrm{H}_{0}:\ p_{control} &\geq p_{treated}\\ \mathrm{H}_{1}:\ p_{control} &< p_{treated}, \end{array} $$

where *p*_*control*_ and *p*_*treated*_ are the true rates in the control and the treatment group, respectively.

All distribution parameters and regression coefficients used to simulate the data are given in Table 1. The simulated data includes three binary variables (incl. the group variable *Z*), one categorical variable, and two continuous variables. The variables are used to simulate the group assignment and the outcome variable, as well as they are considered within the matching procedure.

**Table 1:** Distributions and regression models used for simulating the data

Variable	Group	Distribution	Model
*X*_1_	both	*Bin*(1;0.5)	
*X*_2_	both	*Bin*(1;0.2)	
*X*_3_	both	*N*(70;15)	
*Z*			$logit\left (\frac {P(Z=1)}{1-P(Z=1)}\right)$
			=−0.6+0.35*X*_1_−0.01*X*_3_
*X*_4_	control	*Bin*(10;0.8)	
*X*_4_	treated	*Bin*(10;0.75)	
*X*_5_	control	*N*(17;5)	
*X*_5_	treated	*N*(16;4)	
$Y_{H_{0}}$	both	*Bin*(1;0.5)	
$Y_{H_{1}}$	both		$logit\left (\frac {P(Y=1)}{1-P(Y=1)}\right)$
			=−0.5+*Z*+0.2*X*_4_

First, two binary (*X*_1_,*X*_2_) and one continuous variable (*X*_3_) are sampled which describe for example gender, diabetes (yes/no), and age.

The group assignment depends on the variables *X*_1_ and *X*_3_. Therefore, in the next step, the group variable is simulated based on a logistic regression model using the baseline variables *X*_1_ and *X*_3_.

Based on the group allocation (group is considered as *Z* in the following), two additional variables (*X*_4_ and *X*_5_) are simulated. The variable *X*_4_ is an ordinal variable with 10 levels which represent the ASPECTS score here. The Alberta Stroke Program Early CT score (ASPECTS) is a tool for detecting early ischemic changes on non-contrast CT scans [11]. The variable *X*_5_ follows a normal distribution and describes here the NIHSS. The National Institutes of Health Stroke Scale (NIHSS) is a tool to assess stroke severity [12]. These two additional variables are sampled out of different distributions (according to the group). By using a logistic regression model for the group allocation, as given in Table 1 for variable *Z*, and sampling clinical variables out of different distributions, differences between the groups are simulated which can be addressed by the matching procedure. In order to simplify the simulation study, the variables *X*_1_ to *X*_5_ are assumed to be independent. However, in practice correlations may occure and should be considered when selecting the matching variables.

The propensity score is estimated by using the baseline variables *X*_2_,*X*_3_, and *X*_5_ as covariates in a logistic regression model with group as outcome variable (*Z*). This model includes baseline variable *X*_2_ which is not part of the true group model. This leads to a misspecification of the propensity score model. However, the true model is usually not known and therefore this setting avoids to be overoptimistic in the simulations.

The confidence level is set to *α*_*CI*_∈{0.01,0.05,0.1}, and hence the resampling CI method is evaluated for 99%, 95%, and 90% confidence intervals in this simulation study.

For testing the null-hypothesis at the end of the trial, the McNemar test for paired data is used to account for the matched design.

#### Fixed time point - varying number of control patients

We start with a given number of patients in the control group *n*_*control*_ and a fixed fraction *t* of patients for the interim analysis. We set *t*=0.5 and

$$\begin{array}{*{20}l} n_{control} \in &\{25, 50, 75, 100, 125, 150, 175, 200,\\ &225, 250, 275, 300\}. \end{array} $$

The number of patients needed to show the simulated effect with a power of 80% at a type I error rate of 5% would have been 142 per group. We investigate underpowered as well as overpowered scenarios. Underpowered situtations occure when the expected effect in the existing trial, where our control group is taken from, was higher than expected in the new trial. In cases with a smaller expected effect or multiple primary hypothesis in the existing trial we may face an overpowered scenario. At interim analysis, we calculated the matching rate on *b*=200 resampling sets of size *n*_*treated,interim*_. Using the simulated data as described above and performing the steps listed above, we compare the proposed method with the naive approach. For evaluating the properties of the two approaches, the matching rate at final analysis, the recruited sample size *n*_*treated,final*_, as well as the type I error and power at final analysis were evaluated.

#### Time point of interim analysis

The starting point is a fixed number of patients (in the control group) *n*_*control*_ used as one arm of our controlled trial. The confidence level for the resampling CI method is set to 99%, *α*_*CI*_=0.01 respectively. The “time points” *s* considered for the interim analysis are

$$s \in \left\{ \left\{\frac{1}{10}, \frac{1}{4}, \frac{1}{3}, \frac{1}{2}, \frac{2}{3}, \frac{3}{4}, \frac{9}{10} \right\} \cdot n_{\text{control}} \right\}. $$

For recalculation of the sample size, the resampling CI method as well as the naive approach is used. Our recommendation will be based on the evaluation of the matching rate, the recruited sample size *n*_*treated*_, as well as the type I error and power at final analysis.

We considered a small (*n*_control_=50), medium (*n*_control_=150), and a large (*n*_control_=500) sample size in the control group to identify the influence on the time point of interim analysis. The regression coefficient *β*_*Z,outcome*_ varies between the considered sample sizes to obtain 80% power within each scenario:

$$\begin{array}{*{20}l} \textrm{small: }&\beta_{Z, outcome}= 2\\ \textrm{medium: }&\beta_{Z, outcome}= 1 \\ \textrm{large: }&\beta_{Z, outcome}= 0.55 \end{array} $$

For the small sample size, the considered time points of interim analysis start at $\frac {1}{3} \cdot n_{\text {control}}$ and for the medium sample size at $\frac {1}{4} \cdot n_{\text {control}}$; this is due to problems in finding matching partners if fewer than 15 patients are included in the matching procedure at the interim analysis step.

All simulations were done in R version 3.4.3 with the packages Matching (using the function Match) and boot (using the function inv.logit) [13–15].

## Results

### Simulation results

First, the results using a fixed time point but varying the number of control patients are shown and discussed, followed by the results for the time point of interim analysis which is assessed for both described methods.

#### Fixed time point - varying number of control patients

**Fig. 1:**
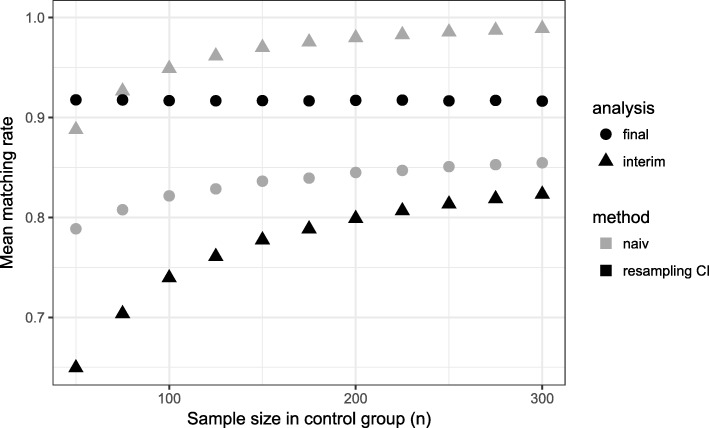
Mean matching rate for different sample sizes in control group. Time point of interim analysis is $\frac {1}{2} \cdot n_{control}$

**Fig. 2:**
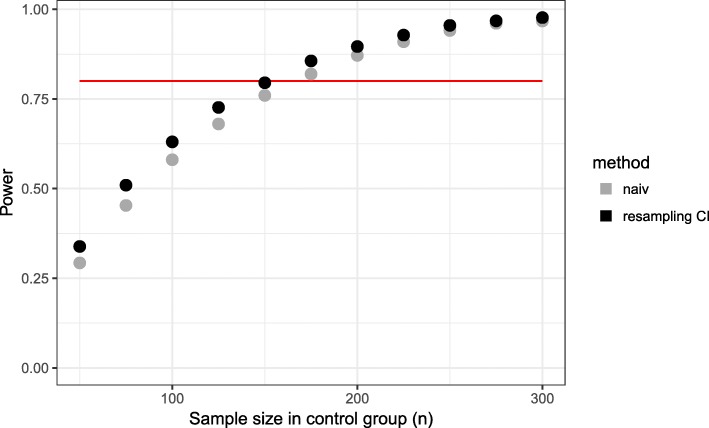
Power for different sample sizes in control group. Time point of interim analysis is $\frac {1}{2} \cdot n_{control}$

Comparing the matching rate curves at interim and final analysis within the naive approach, one observes that the matching rate at interim analysis is higher in all scenarios. The consequence of an overestimation of the matching rate at interim analysis is the recruitment of a too small number of patients. If the matching rate is smaller at final analysis (overestimation of matching rate at interim analysis), this results in a loss of power (Figs. 1, 2).

**Fig. 3:**
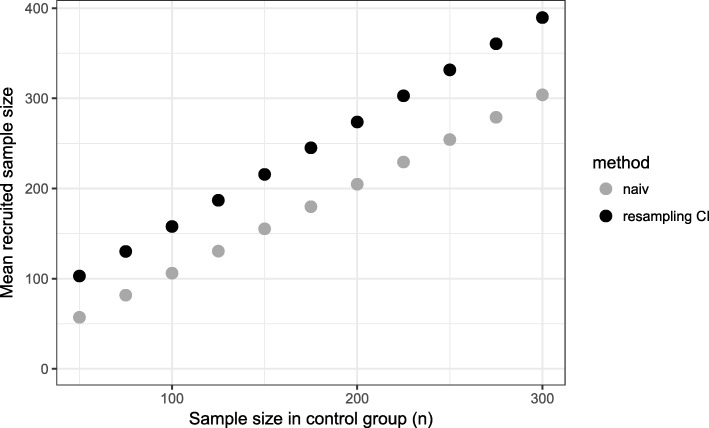
Mean sample size in treated group for different sample sizes in control group. Time point of interim analysis is $\frac {1}{2} \cdot n_{control}$

Our proposed method uses equal sample sizes for the matching procedure at interim analysis which underestimates the true matching rate. Therefore, more patients are recruited (Fig. 3) and a higher matching rate is achieved at the final analysis. Hence, too much matched pairs are included in the final analysis which results in a higher power.

For the naive method, we observed a dependency between the matching rate and the number of patients in the control group: the matching rate grows with the number of patients in the control group. In contrast, for our proposed method the matching rate at final analysis stays on a constant level with a mean matching rate between 91.7 - 91.8% for *α*_*CI*_=0.01

Here, the required number of patients per group in a fixed design would have been *n*=142. For the proposed design, 80% power is reached for *n*_*control*_≈150. Thus, a power of 80% is achieved requiring only slightly more patients in the control group than would have been in a fixed randomized design (Fig. 2). This higher number of control patients is caused by the matching rate which is lower than 100% (Fig. 1).

**Fig. 4:**
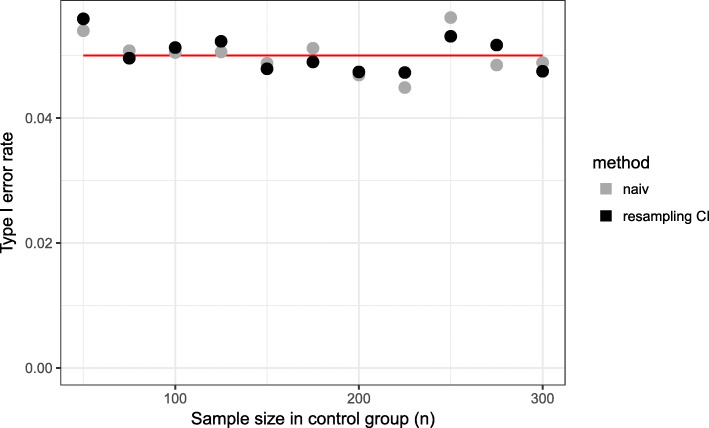
Type I error rate for different sample sizes in control group. Time point of interim analysis is $\frac {1}{2} \cdot n_{control}$

Type I error rate is approximately 5% (between 4.37% and 5.72%) for all scenarios and both methods. As expected, a difference between the two methods according to the type 1 error is not observed (Fig. 4).

Varying the confidence level within the resampling CI method results in small differences in the mean lower CI limit of the matching rate. The mean lower CI limit of the matching rate at interim analysis increases slightly for increasing *α*_*CI*_ or decreasing confidence level, respectively (Table 2). This increase leads to a slightly lower number of recruited patients for lower confidence levels. For *α*_*CI*_=0.05, the mean recruited sample size in the treatment group is around 4 patients higher than for *α*_*CI*_=0.1, and for *α*_*CI*_=0.01 is for another 8 patients higher, for details see Table 3.

**Table 2:** Mean matching rate/mean lower CI limit of the matching rate at interim and final analysis for the naive approach and the resampling CI method for different numbers of patients in the control group

	Interim	Final	Interim	Final
*n*_*control*_	Naiv		99%-CI	
50	0.89	0.79	0.49	0.92
75	0.93	0.81	0.58	0.92
100	0.95	0.82	0.64	0.92
125	0.96	0.83	0.67	0.92
150	0.97	0.84	0.70	0.92
175	0.98	0.84	0.72	0.92
200	0.98	0.84	0.73	0.92
225	0.98	0.85	0.74	0.92
250	0.99	0.85	0.76	0.92
275	0.99	0.85	0.76	0.92
300	0.99	0.86	0.77	0.92
	95%-CI		90%-CI	
50	0.54	0.91	0.56	0.90
75	0.62	0.91	0.64	0.90
100	0.67	0.91	0.68	0.91
125	0.70	0.91	0.71	0.91
150	0.72	0.91	0.73	0.91
175	0.74	0.91	0.75	0.91
200	0.75	0.91	0.76	0.91
225	0.76	0.91	0.77	0.91
250	0.77	0.91	0.78	0.91
275	0.78	0.91	0.79	0.91
300	0.79	0.91	0.80	0.91

The resampling CI method is applied for different confidence levels (*α*_*CI*_∈{0.01,0.05,0.1})

**Table 3:** Mean total number of recruited patients in the treament group for the naive approach and the resampling CI method for different numbers of patients in the control group

*n*_*control*_	Naiv	99%-CI	95%-CI	90%-CI
50	57.20	103.05	94.11	89.97
75	81.88	130.51	122.83	119.10
100	106.24	158.13	150.99	147.45
125	130.73	187.46	180.42	176.88
150	155.34	215.80	208.85	205.32
175	179.98	245.10	238.09	234.52
200	204.84	273.86	266.81	263.19
225	229.42	303.28	296.11	292.42
250	254.39	331.75	324.53	320.80
275	279.19	361.06	353.69	349.90
300	304.00	389.50	382.07	378.21

#### Time point of interim analysis

In this section, we only consider the results for a medium sample size in the control arm (*n*_control_=150), as the simulations for small and large sample sizes show comparable results.

**Fig. 5:**
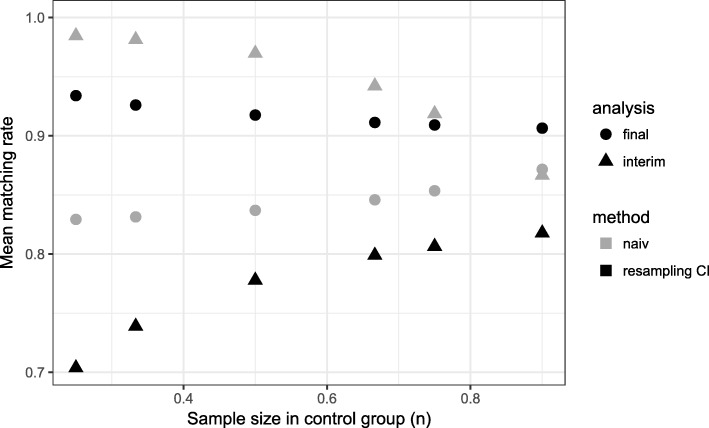
Mean matching rate for different time points of the interim analysis (*n*_*control*_=150)

**Fig. 6:**
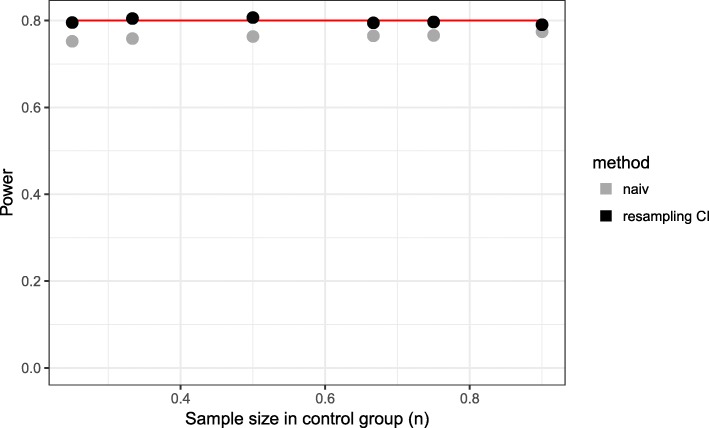
Power for different time points of the interim analysis (*n*_*control*_=150)

**Fig. 7:**
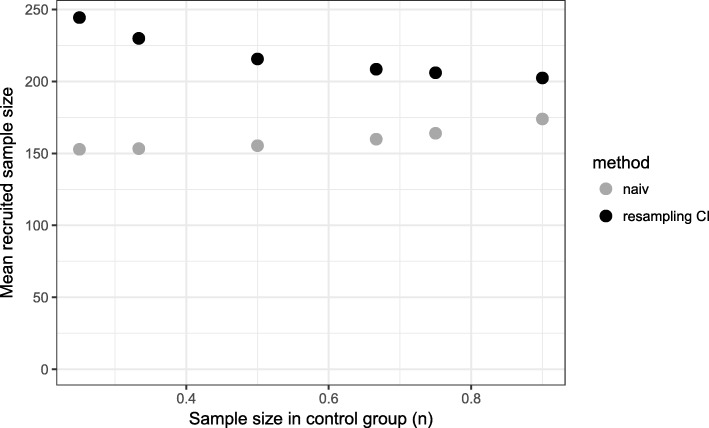
Mean sample size in treated group for different time points of the interim analysis (*n*_*control*_=150)

**Fig. 8:**
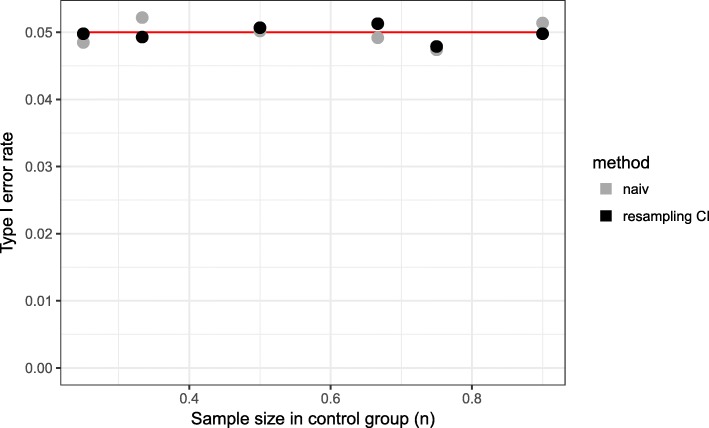
Type I error for different time points of the interim analysis (*n*_*control*_=150)

Using the naive method, we observe for early time points of the interim analysis a matching rate close to 100%, but in the final analysis, it is less than 85% (Fig. 5). Even for later time points, the matching rate is lower than 90% and as a consequence the power is less than 80% for all considered time points (Fig. 6). The total sample size is lowest for the early time point (Fig. 7) because the matching rate at interim analysis is highest for this time point. As expected, the type 1 error rate is around 5% (Fig. 8).

Our proposed method uses equal sized groups at the interim analysis. When performing an early interim analysis, the matching rate is very low and a high number of patients need to be additionally recruited for the final analysis. Comparing the matching rate at the final analysis between the different time points, the gain in the matching rate and therefore in power is small when performing an early interim analysis. With an increasing number of patients at interim analysis the matching rate seems to converge and the changes are very small (in the matching rate) when increasing the number of patients in the control group used at interim analysis above 50% (Fig. 5). Taking also the recruited sample size into account, it seems that a time point between $\frac {1}{2}$ and $\frac {2}{3}$ of the control patients is a good choice as a trade-off between matching rate and sample size (Fig. 7). The matching rate lies between 90.7% and 93.4% for all considered time points. In all scenarios, the achieved power is around 80% and the type I error rate around 5% (Figs. 6, 8).

It seems that for small sample sizes in the control group a later interim analysis could be a good choice and for large sample sizes an earlier time point, respectively. Using only 50% of the control patients at the interim analysis in small trials leads to a low absolute number of patients which underestimates the matching rate. For large sample sizes, it is observed that a smaller absolute number of control patients leads to a good estimate of the matching rate. The results are shown in the Additional files 1 and 2: (Figures S1 to S8).

### Real data example

The KEEP SIMPLEST trial aimed to compare aspects of periinterventional management in AIS patients treated according to a new SOP (standard operating procedure) with patients having been randomized into the conscious sedation group of the SIESTA trial [7].

The CS group of the SIESTA trial includes 77 patients but only 73 were considered in the matching analysis due to missings in matching variables. The study protocol intended an interim analysis after 50 patients to estimate the matching rate. The actually recruited number of treated patients at interim analysis was 51. We applied the resampling CI method for the recalculation of sample size using 200 resampling steps. To compare the two methods here, we additionally conducted the analysis using the naive method.

Within the matching procedure, four baseline variables were considered: Age, NIHSS on admission, premorbid mRS, and the ASPECTS score. The propensity score was estimated by a logistic regression model. For the propensity score matching, a caliper width of 0.2 of the standard deviation of the propensity score was used. The interim analysis showed the following results:

$$\begin{array}{*{20}l} mr_{naiv} =& 0.607\\ \overline{mr} =& 0.461 \end{array} $$

The extrapolation of $\overline {mr}=0.461$ resulted in a total sample size of 161. The KEEP SIMPLEST data set consists of 154 patients with complete data (161 patients were included in the trial but 7 patients had a missing ASPECTS score). The matching procedure reached 0.945 matching rate in the final analysis, hence 69 pairs were found and analyzed.

The naive method would result in a total sample size of 122. Using only 122 patients in the treated group for the second matching procedure would have resulted in 63 matched pairs and hence a matching rate of 0.863. Thus, in our real data example, the resampling CI method achieves a 8.2% higher matching rate in the final analysis.

## Discussion and conclusion

In this article, we propose to include an interim analysis step within a prospective matched case-control trial. We showed by simulations that the naive method might severly overestimate the matching rate at the interim analysis. This leads to a low matching rate at the final analysis and, therefore, a too low power. The resampling CI method avoids this overestimation and the recalculation results in a higher number of treated patients. As a consequence, a higher matching rate can be achieved at the final analysis and this is related to a gain in power. At the same time this approach still leads to a reasonable sample size and is therefore a very efficient approach. An increase of the confidence level showed only a small influence on the sample size in the treatment group and matching rate at final analysis. The application to a real data example also showed that the resampling CI method leads to a very good matching rate in contrast to the naive approach.

Our proposed approach is a powerful technique to reach a good matching rate and a high power at the final analysis. The simulation study demonstrates the characteristics of the approaches based on a single model including different types of covariates. Even though more complex models are not evaluated in the simulations, a higher model complexity is not expected to strongly influence the performance of the approaches when model convergence is guaranteed. A higher degree of misspecification of the propensity score model would lead to a lower matching rate. However, this would be the case for both methods. A limitation of our approach is the very specific application area and the relatively high number of intervention patients to be included. Another limitation is the fact that the maximal sample size per group is limited by the number of patients in the control group which leads to power restrictions. On the other hand, in case a very large control group exists, for instance out of a registry, our proposed method might underestimate the true matching rate at interim analysis and one should consider a 1:*k* matching design. As a trade-off between matching rate, power, and sample size, we recommend a proportion of $\frac {1}{2}$ to $\frac {2}{3}$ of the number of patients in the control group at the interim analysis. It appears that it depends more on the absolute number of patients at the interim analysis than on the relative number of control patients to get a good estimate for the matching rate. Giving a recommendation for an absolute number of patients needed for the interim analysis independent of the trial size is difficult, because this number would be limited by the trials with a small sample size. Nevertheless, we provide clear recommendations for situations that appear typical in clinical trials and therefore helpful instructions for clinicial researchers in the planning stage of a trial.

## Additional files

Additional file 1 Time point of Interim Analysis - Small Sample Size. **Figure S1** Mean matching rate for different time points of the interim analysis (*n*_*control*_=50). **Figure S2** Power for different time points of the interim analysis (*n*_*control*_=50). **Figure S3** Mean sample size in treated group for different time points of the interim analysis (*n*_*control*_=50). **Figure S4** Type I error for different time points of the interim analysis (*n*_*control*_=50). (ZIP 17.9 kb)

Additional file 2 Time point of Interim Analysis - Large Sample Size. **Figure S5** Mean matching rate for different time points of the interim analysis (*n*_*control*_=500). **Figure S6** Power for different time points of the interim analysis (*n*_*control*_=500). **Figure S7** Mean sample size in treated group for different time points of the interim analysis (*n*_*control*_=500). **Figure S8** Type I error for different time points of the interim analysis (*n*_*control*_=500). (ZIP 17.6 kb)

## Data Availability

Any requests for simulated data should be directed to the corresponding author. The data of the real data example are not publicly available as it may contain information that could compromise research participant privacy.

## Abbreviations

AIS: Acute ischemic stroke
ASPECT: Alberta stroke program early CT
CI: Confidence interval
NIHSS: National institutes of health stroke scale
SOP: Standard operating procedure
