# Supplementary figures and images for: Adaptive propensity score procedure improves matching in prospective observational trials
Source: BMC Med Res Methodol. 2019 Jul 16;19:150. doi: 10.1186/s12874-019-0763-3 (PMC6636117; doi:10.1186/s12874-019-0763-3)

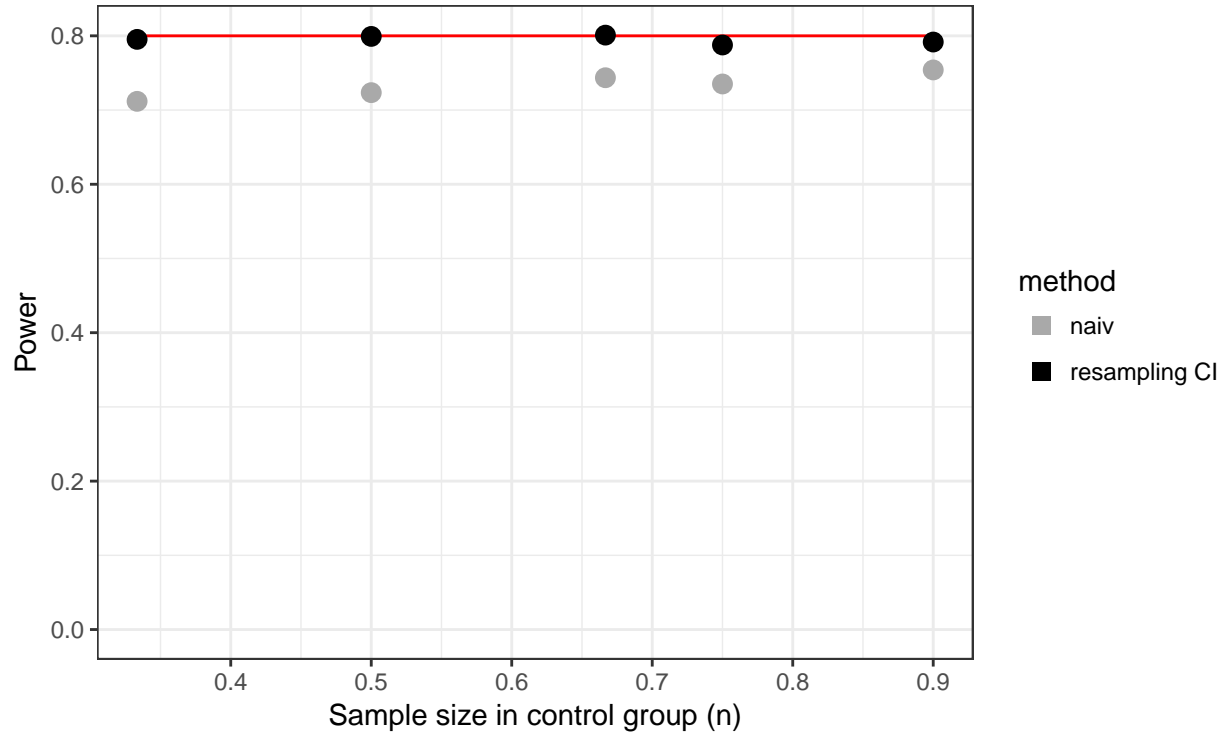

Supplement: Supplementary file 1 — Time point of Interim Analysis - Small Sample Size. Figure S1 Mean matching rate for different time points of the interim analysis (ncontrol=50). Figure S2 Power for different time points of the interim analysis (ncontrol=50). Figure S3 Mean sample size in treated group for different time points of the interim analysis (ncontrol=50). Figure S4 Type I error for different time points of the interim analysis (ncontrol=50). (ZIP 17.9 kb) [file 12874_2019_763_MOESM1_ESM.zip › Additional file 1/Figure10R1.pdf]

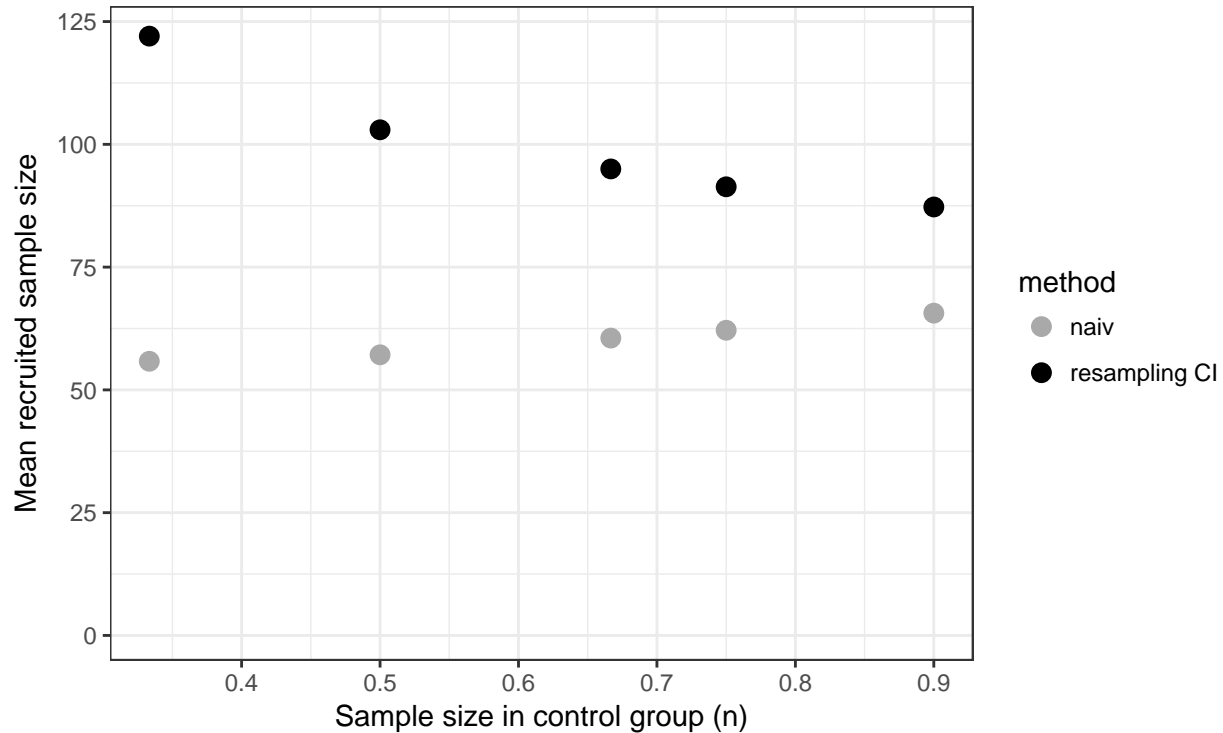

Supplement: Supplementary file 1 — Time point of Interim Analysis - Small Sample Size. Figure S1 Mean matching rate for different time points of the interim analysis (ncontrol=50). Figure S2 Power for different time points of the interim analysis (ncontrol=50). Figure S3 Mean sample size in treated group for different time points of the interim analysis (ncontrol=50). Figure S4 Type I error for different time points of the interim analysis (ncontrol=50). (ZIP 17.9 kb) [file 12874_2019_763_MOESM1_ESM.zip › Additional file 1/Figure11R1.pdf]

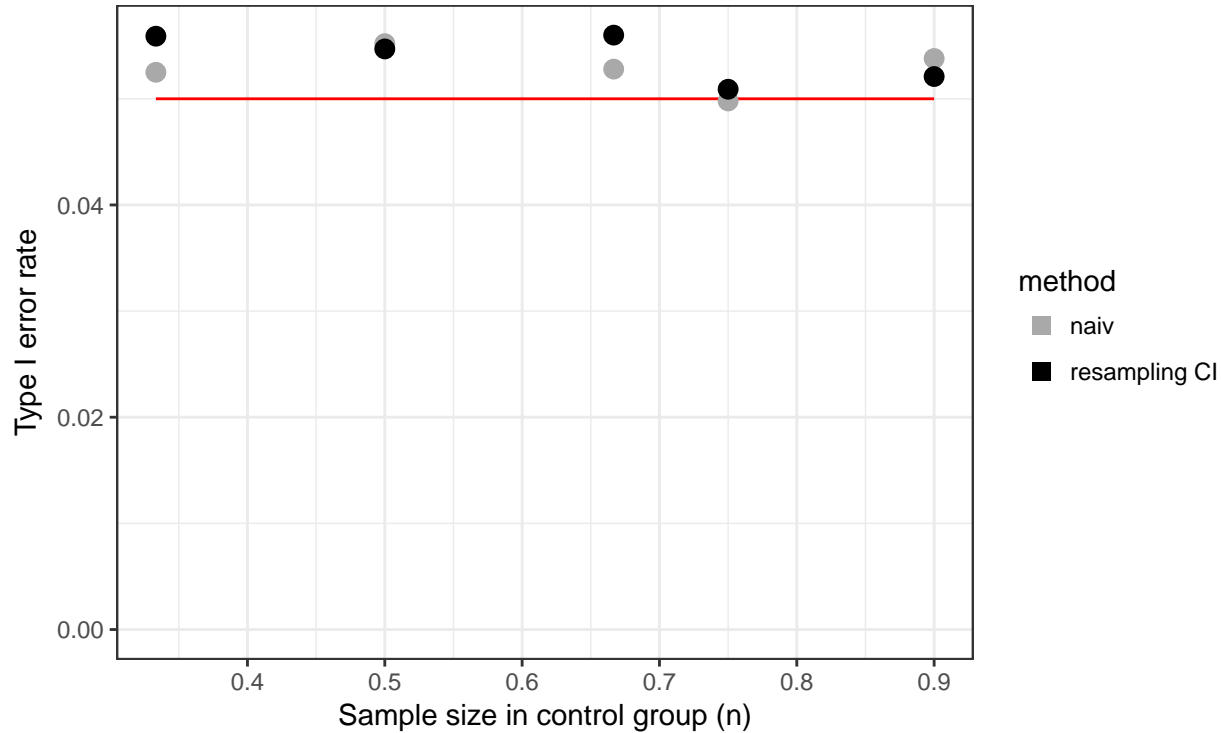

Supplement: Supplementary file 1 — Time point of Interim Analysis - Small Sample Size. Figure S1 Mean matching rate for different time points of the interim analysis (ncontrol=50). Figure S2 Power for different time points of the interim analysis (ncontrol=50). Figure S3 Mean sample size in treated group for different time points of the interim analysis (ncontrol=50). Figure S4 Type I error for different time points of the interim analysis (ncontrol=50). (ZIP 17.9 kb) [file 12874_2019_763_MOESM1_ESM.zip › Additional file 1/Figure12R1.pdf]

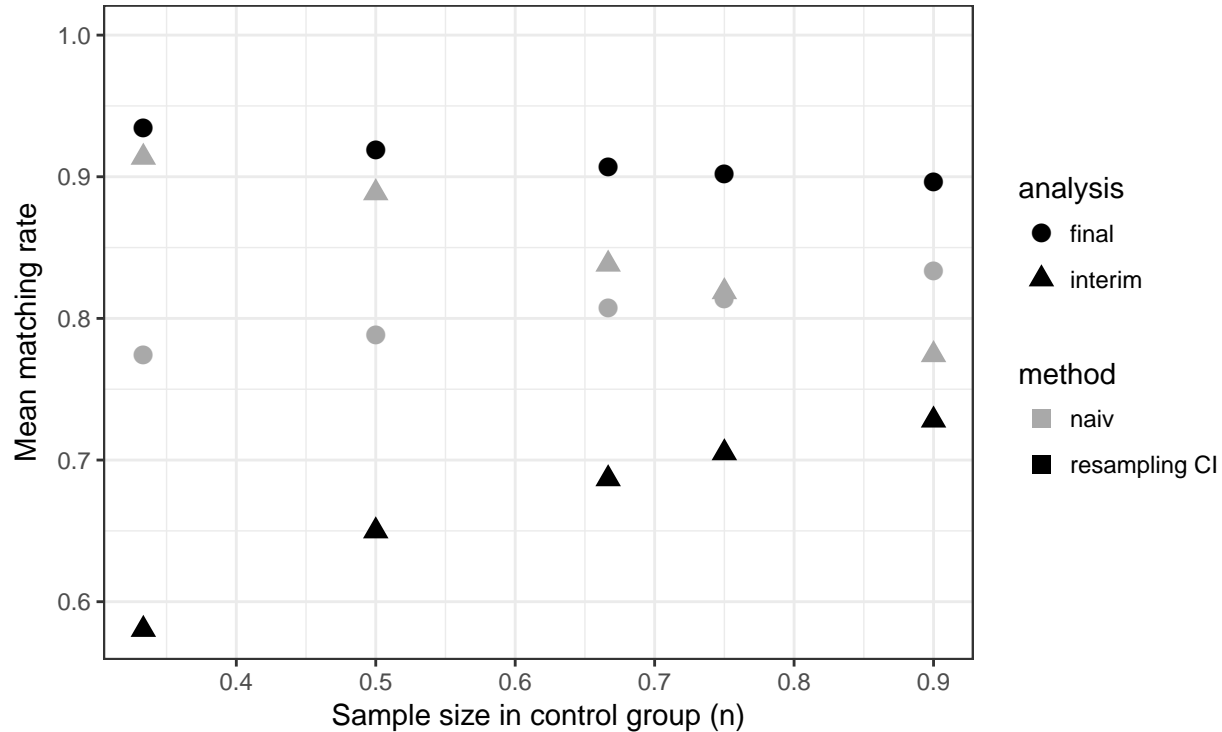

Supplement: Supplementary file 1 — Time point of Interim Analysis - Small Sample Size. Figure S1 Mean matching rate for different time points of the interim analysis (ncontrol=50). Figure S2 Power for different time points of the interim analysis (ncontrol=50). Figure S3 Mean sample size in treated group for different time points of the interim analysis (ncontrol=50). Figure S4 Type I error for different time points of the interim analysis (ncontrol=50). (ZIP 17.9 kb) [file 12874_2019_763_MOESM1_ESM.zip › Additional file 1/Figure9R1.pdf]

Mean matching rate

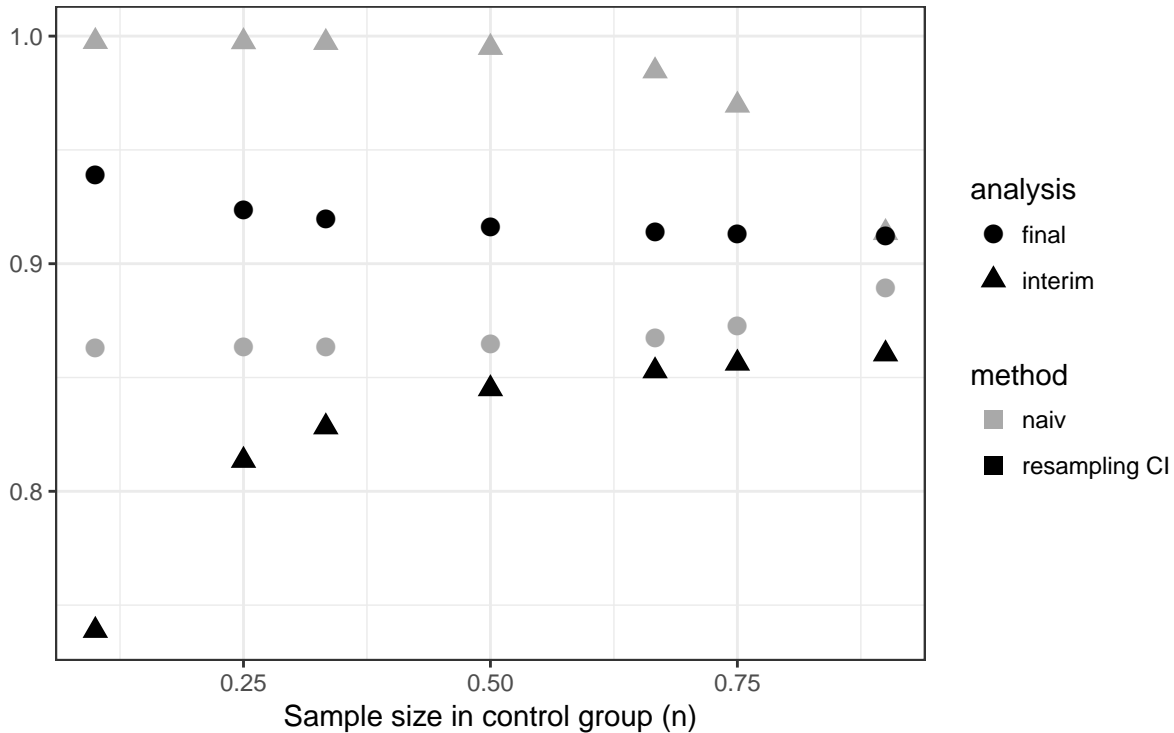

Supplement: Supplementary file 2 — Time point of Interim Analysis - Large Sample Size. Figure S5 Mean matching rate for different time points of the interim analysis (ncontrol=500). Figure S6 Power for different time points of the interim analysis (ncontrol=500). Figure S7 Mean sample size in treated group for different time points of the interim analysis (ncontrol=500). Figure S8 Type I error for different time points of the interim analysis (ncontrol=500). (ZIP 17.6 kb) [file 12874_2019_763_MOESM2_ESM.zip › Additional file 2/Figure13R1.pdf]

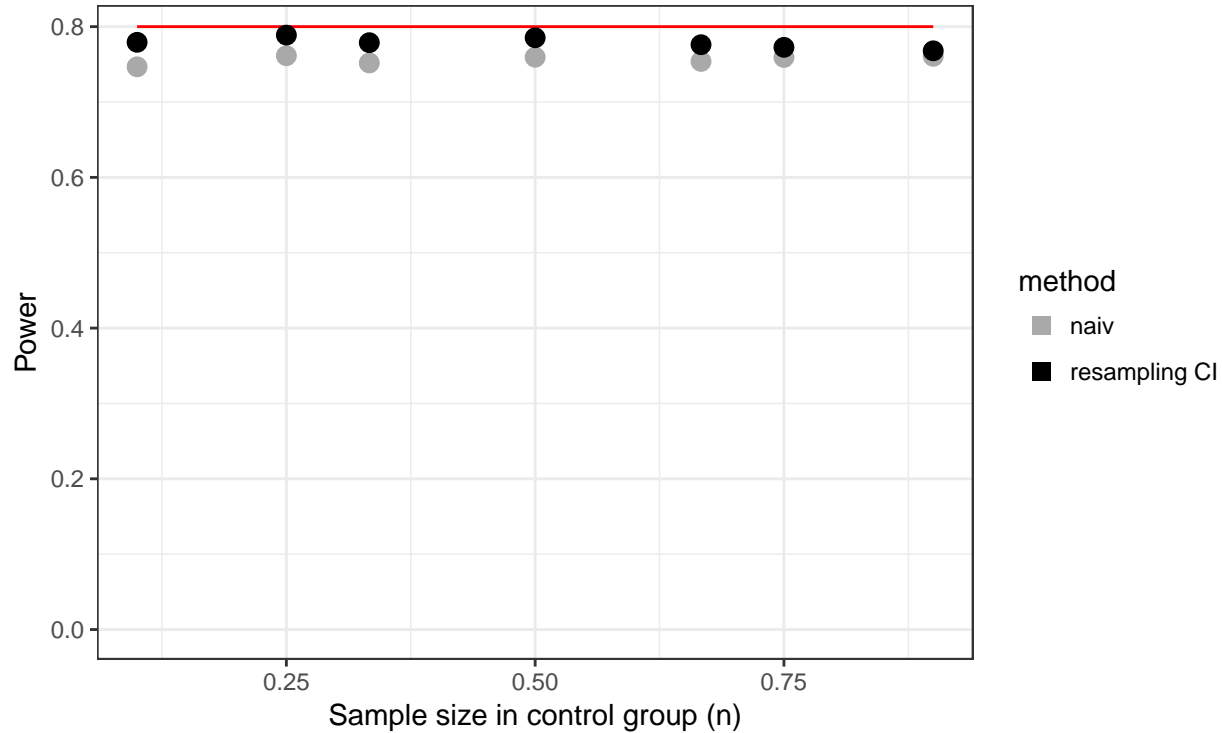

Supplement: Supplementary file 2 — Time point of Interim Analysis - Large Sample Size. Figure S5 Mean matching rate for different time points of the interim analysis (ncontrol=500). Figure S6 Power for different time points of the interim analysis (ncontrol=500). Figure S7 Mean sample size in treated group for different time points of the interim analysis (ncontrol=500). Figure S8 Type I error for different time points of the interim analysis (ncontrol=500). (ZIP 17.6 kb) [file 12874_2019_763_MOESM2_ESM.zip › Additional file 2/Figure14R1.pdf]

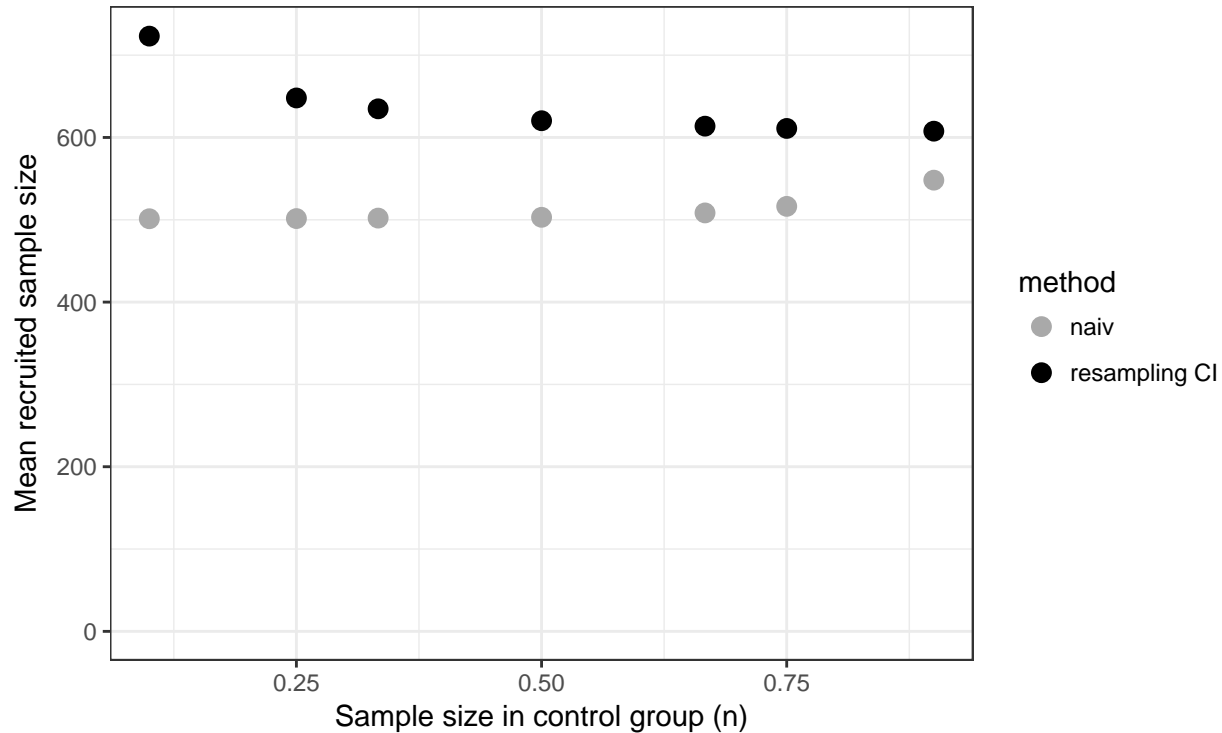

Supplement: Supplementary file 2 — Time point of Interim Analysis - Large Sample Size. Figure S5 Mean matching rate for different time points of the interim analysis (ncontrol=500). Figure S6 Power for different time points of the interim analysis (ncontrol=500). Figure S7 Mean sample size in treated group for different time points of the interim analysis (ncontrol=500). Figure S8 Type I error for different time points of the interim analysis (ncontrol=500). (ZIP 17.6 kb) [file 12874_2019_763_MOESM2_ESM.zip › Additional file 2/Figure15R1.pdf]

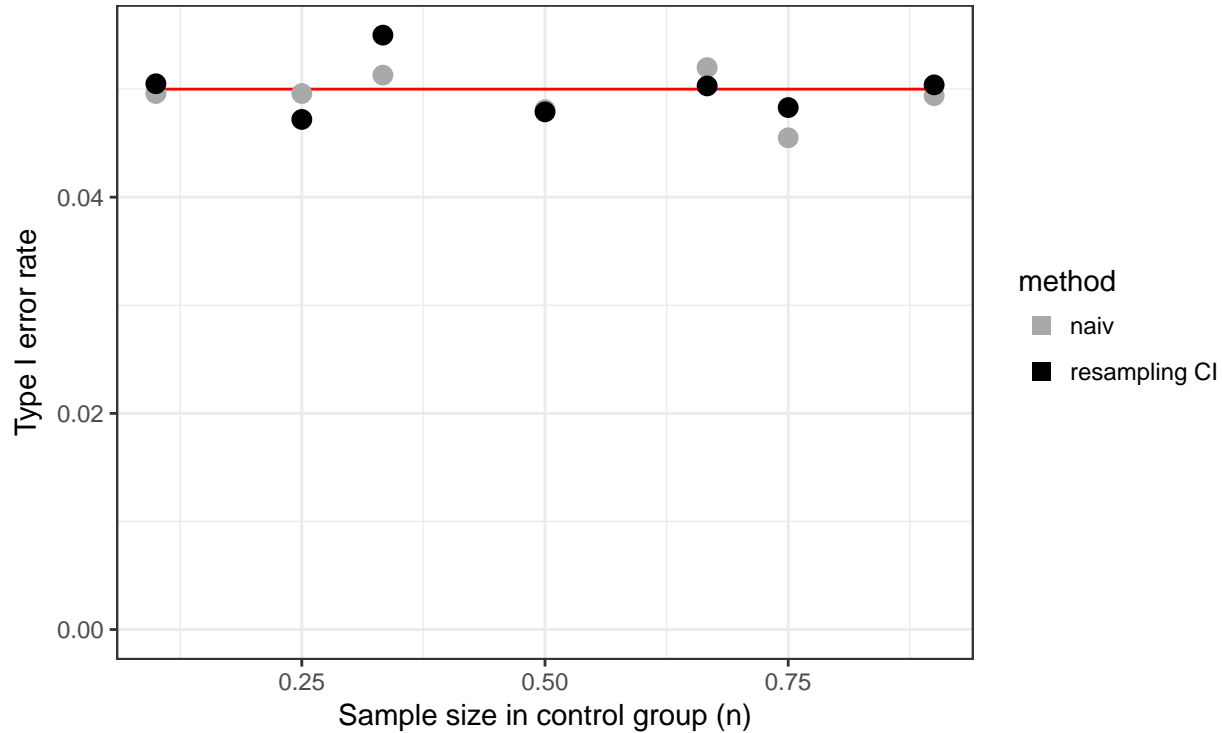

Supplement: Supplementary file 2 — Time point of Interim Analysis - Large Sample Size. Figure S5 Mean matching rate for different time points of the interim analysis (ncontrol=500). Figure S6 Power for different time points of the interim analysis (ncontrol=500). Figure S7 Mean sample size in treated group for different time points of the interim analysis (ncontrol=500). Figure S8 Type I error for different time points of the interim analysis (ncontrol=500). (ZIP 17.6 kb) [file 12874_2019_763_MOESM2_ESM.zip › Additional file 2/Figure16R1.pdf]
